# Supplementary material for: Patterns of Intron Gain and Loss in Fungi
Source: PLoS Biol. 2004 Nov 30;2(12):e422. doi: 10.1371/journal.pbio.0020422 (PMC532390; doi:10.1371/journal.pbio.0020422)
Supplement: Table S1 — Also available at http://genes.mit.edu/NielsenEtAl/. (4.3 MB ZIP). [file pbio.0020422.st001.zip › NielsenEtAl/html/301.html]

AN2900.1.NCU09025.1.MG03390.1.FG06078.1


```
 CLUSTAL W (1.82) Multiple Sequence Alignments - Introns Inserted


Sequence 1: NCU09025.1	647 aa
Sequence 2: FG06078.1	617 aa
Sequence 3: MG03390.1	654 aa
Sequence 4: AN2900.1	602 aa
Alignment Length: 678 aa
Number Identitical Residues: 222 aa
Alignment Score (without introns) 11392


MG03390.1 	MGRIPTRELSSYLLTGRVFSRDCTR--------LQRLPARNISKQHFAVGQPAQRRWNHT
NCU09025.1	MPRIPTVELSRYLLTGHIASPGCLRARAALPGKNTRVALGGAPLPSRQLQPRQHARYLTT
FG06078.1 	MSRIPTSILSKYLLKGQIPRQGCIG---------THHISRHLALTSAAASLAHTTRHLHT
AN2900.1  	MFRS----CLRHCRRATVRSRTCPR--------CSHHEIPQLQVVQRQISLSSSFPHIRR
          	* *        :   . :    *            :                        

MG03390.1 	APEAGHTTTTQPPPGQPAPHLRKILKDQAKASKAQAKGKKKRSSADNQTVPGWELTVGIE
NCU09025.1	ETSFANPSATTSSPAAAAP-FRKQLKEQAKALKKS--GHKKKKSSDNQTVPGWELTVGIE
FG06078.1 	S-----PEVITPPPVQPVP-LRKQLKDEAKKAKKQ--GKKKIKG-DSQTVDGWELTVGIE
AN2900.1  	L------QTSSTDTQERIP-IRKQLKQEAKAIKSR---KRERREQEEASRHKWELTVGVE
          	        .  . .    * :** **::**  *     :::    :. :   ******:*

MG03390.1 	IHAQLNTDRKLFSP~APLASTEDNTRAPAPNSLVAPFDLAIPGTQP~LFQQATLVPAIRA
NCU09025.1	IHAQLNTAHKLFSP~ATTSFNDP------PNTHVAPFDLALPGSQP~LFQPATLIPAVRA
FG06078.1 	IHAQLNTAHKLFSP~AITSFNDE------PNSHVALFDVAMPGSQP~LFQKETLIPALRA
AN2900.1  	IHAQLNTETKLFSR1APTSPSEL------PNTNVALFDLAFPGSQP0EFQVATLLPALRA
          	*******  ****  *  : .:       **: ** **:*:**:**  **  **:**:**

MG03390.1 	AVALNCAVQPVSSFDRKHYFHWDQPSGYQITQYYA~PLARDGYVDLRARDGIAAQDVGGG
NCU09025.1	ALALNCSIQPVSRFDRKHYFHWDQPSGYQITQFYE~PFAKDGFITLYARDGIAAED---G
FG06078.1 	ALSLNCEVQEVSRFDRKHYFWWDQPSGYQITQYYE~PFAKNGHITLLARDGISPQD---G
AN2900.1  	AIALNCDIQPVSRFDRKHYFYQDQPSGYQITQYYE1PFARNGYLDLFRHDGIAPED---G
          	*::*** :* ** *******  **********:*  *:*::*.: *  :***:.:*   *

MG03390.1 	EPLRIRVKQVQMEQDTAKTLARPDGVHWLDFNRCGAPLVEIISEPDIHHPATAAAFVRKV
NCU09025.1	EEIKVGIKQVQMEQDTAKTTAQPGDVQWLDFNRVGVPLIEIITLPEIHHPATAAALVRKV
FG06078.1 	ESVTIGIKQVQLEQDTAKTLAQTDKVQWLDFNRVGVPLIEIITEPELHHPRTAAVLVRKI
AN2900.1  	DRVRIGIKQIQLEQDTAKSQEYPPSMQLLDFNRVSHPLIEIITMPEIHTPATAAAFVRKV
          	: : : :**:*:******:   .  :: ***** . **:***: *::* * ***.:***:

MG03390.1 	QMLLGAADACVVGMEKGGLRADVNVSVRRVEDGK-----TSSAKLGQRTEIKNLFSFKAV
NCU09025.1	QMVLASVDACVSGLEEGGLRADVNVSVRRTDD--------PSGKLGTRTEIKNLSSFKAV
FG06078.1 	QMLLNTVDACVSGMETGGLRADVNVSVRRTDG--------SNPSLGTRTEIKNLSTIKAV
AN2900.1  	QAILQSCSAVTTGMEAGGLRADINVSVRLRGDGSGTHQYSGIGGLGQRTEIKNLSSFKAV
          	* :* : .* . *:* ******:*****   ....: . :    ** ******* ::***

MG03390.1 	EDAIIAERDRQIKLLDEGGVVLGETRGWSLGSTETRRLRGKEGEVDYRYMPDPDLGPVLV
NCU09025.1	EDAIIAERDRQIELLEEGGEVKGETRGWSLGSTETRRLRGKEGEVDYRYMPDPDLGPVVI
FG06078.1 	EDAIISERDRQIRELEAGRSIPSETRGWSIGSTETRRLRGKEGEVDYRYMPDPDIAPLVI
AN2900.1  	EDAIIAEKNRQIAVLESGGVVEGETRGWTIGSTETRKLRGKEGEVDYRYMPDPDIPPLLI
          	*****:*::***  *: *  : .*****::******:*****************: *:::

MG03390.1 	GQDVVEHLKSTMGVMPDQELDDLVSSYGLSEKDAMSLMLLEDGGRLQFYYKTVDALEQRL
NCU09025.1	GEDLVARLRETMGVLPDQEADQLMERYNLSAKDALSLMLLDGGARIQYFYNVLDSLEERL
FG06078.1 	GNDLVGHLRQSLAVTPDSELDTLIAQFGLTAKDALSLINLENGSRVQYYYKVLYSIQEKL
AN2900.1  	GKDIISALSNTLPAGPDALIDMLVGQYGLAIEDAKPLVELEDGARLEYYQDVVDILRNLQ
          	*:*::  * .:: . **   * *:  :.*: :** .*: *:.*.*:::: ..:  :.:  

MG03390.1 	QNQGDSTKVTAESLVQARILTGNWILHVLGSLTSENNQRDRAAAGLSERDLGVTSEGD-A
NCU09025.1	VADG--QAVPEGAEHAT--LAANWCLHELGKLTDSASSSS-CSDPDVLEGLAMTPLGESP
FG06078.1 	AENMGETEMPEFKSYSA--VVGNWIIHELGRLTTYKAG------PLAARDLAFTPEGDCE
AN2900.1  	QDLDSKTQAGLGR------VAGNWVLHELGGLLS-------------KAGLAW----DAE
          	    ..             :..** :* ** *                 .*.     :. 

MG03390.1 	FISADDLADILFFLHTSRIRQGTAKDLLFAMFNEVMPAQYISTPAGIERYITDNDLWFSE
NCU09025.1	LVPSSSLAAILHHLYSRTITAKVAKDLLWAVYRGEIP------EGGTTSYIDTHGLWFKE
FG06078.1 	QVPDSDLSQLLYHLIRKQITGKVAKELLFAIYLKEI-------DGGVTQAIEDNDLWFKE
AN2900.1  	RVTAESLAALIDQLQRKRITGATAKKVLAMLFDGDRR--------PVAQLLEEENLILRP
          	 :. ..*: ::  *    *   .**.:*  ::                  :  ..* :  

MG03390.1 	LSPQEYSELAESVLDEEEHVLKEFMGARY------PQGKLMFLVGMMMKSGARERIDPAT
NCU09025.1	LPEEEYAKLADEVIQGEEKILGEFLRWKQGKMKAYPQGKLMFLVGKMMRGGPEGRVEASG
FG06078.1 	ISVEEYEQLADEAMEGEDKILQEFANSEQ-----FPQGKLMFLVGKMMRLGPTERIVPAN
AN2900.1  	LSREEYIALASAAIELNPQMVEQIRSKNQ-------LGKLGWFVGQMMRMGEKGRVEAQK
          	:. :**  **. .:: : ::: ::   .         *** ::** **: *   *: .  

MG03390.1 	AQKVMRDVVETRVAAMK----
NCU09025.1	AERVLRRRIEEVYLPELEKGE
FG06078.1 	AERVMRAKVETLRRT------
AN2900.1  	ADAILRELILGLSQ-------
          	*: ::*  :
```
